# The ecological footprint of *Acca sellowiana* domestication maintains the residual vertebrate diversity in threatened highlands of Atlantic Forest

**DOI:** 10.1371/journal.pone.0195199

**Published:** 2018-04-04

**Authors:** Juliano André Bogoni, Maurício Eduardo Graipel, Nivaldo Peroni

**Affiliations:** 1 Programa de Pós-Graduação em Ecologia, Departamento de Ecologia e Zoologia, Universidade Federal de Santa Catarina, Florianópolis, Santa Catarina, Brazil; 2 Departamento de Ecologia e Zoologia, Universidade Federal de Santa Catarina, Florianópolis, Santa Catarina, Brazil; University of New England, AUSTRALIA

## Abstract

Past and contemporary human actions are causing numerous changes in patterns and processes at various ecosystem scales and trophic levels, including unintended downstream changes, such as species interactions. In its native range *Acca sellowiana* (Feijoa) combines some characteristics of human interactions: incipient domestication, restricted to subtropical Atlantic Forest highlands, associated with the threatened conifer *Araucaria angustifolia* (Araucaria), within a domesticated landscape with anthropogenic forest patches, and provides fruit at a time of resource shortage (Araucaria seeds—*pinhão*). We quantify the trophic relationships between Feijoa and vertebrates, and evaluate the influences on interactions caused by environmental variations, Feijoa domestication evidences, spatial distance and fruit availability. In four sites within protected areas, we selected 28 focal individuals of Feijoa (seven/site) and collected three temporal replicas between 2015 and 2016, when we measured productivity and frugivory via 45-second videos taken with camera traps. Using ecological network, rarefaction curves and variation partitioning analyses, we evaluate the frugivory network topology, the spatiotemporal structure of communities in relation to fruit availability and the influence of predictive variables on frugivory. We found a large spatiotemporal variation in productivity of Feijoa and that 20 species consumed Feijoa fruits, with a species degree of 2.8 (±5.7) and average Feijoa degree of 14.4 (±10.1), in a modular network with intermediary connectance. Rarefaction curves showed that richness and the independent records are congruent with the fruit amount. Variation partitioning showed that, for the focal individuals, canopy area, green coverage, patch size and distance to water influenced frugivory, and the Feijoa domestication influenced significantly the mammalian frugivory. Feijoa is an important resource that provides food during the time of year when *Pinhão* is absent, and attracts frugivores, maintain the residual diversity of vertebrates contributing to the structure of communities in highlands. Our insights allowed us to evaluate the magnitude of the interactions between vertebrates and an incipient domesticated tree, in a cultural landscape and highly threatened environment, under a basal foodweb approach with implications for bottom-up and top-down forces. The results contribute to understanding animal-plant relationships, including concepts that can be replicated for other sessile prey and mobile predators in any region or habitat under different gradients of management. Thus, this work shows how human actions can change not only patterns of distribution and abundance but also the diversity and direction of interspecific interactions among species.

## Introduction

Ecological systems are complex, dynamic, self-organized, with emergent properties, and feedbacks [1]. In addition, past changes and the persistent feedbacks generated by human ecosystem engineers in coconstructed landscapes with nature have important implications for current diversity patterns and ecological processes [2–3]. Yet, contemporaneous disruptions can promote numerous changes and move ecosystems from one stable state to another stable alternative: a process known as phase shifts [4]. One of these disorders is “Anthropocene Defaunation”: a process of extinction caused by human impacts [5–6]. The main human-driven causes of defaunation and compositional changes are habitat loss, fragmentation, hunting and forest conversion [7–8]. Compositional changes and selective species loss in communities can produce strong side-effect impacts on ecosystem function, promoting cascade effects at several scales [9]. These effects include evolutionary changes (e.g., decreasing seed size) and affect population dynamics and community structure (e.g., changes in the detritivore foodweb, seed dispersal and trophic webs, and rearrangements of top-down and bottom-up forces) [9–11].

The top-down model of community regulation predicts that organisms can be limiting resources, both as predator or prey, depending on their position in the food web [12]. The effectiveness of top-down vs. bottom-up forces in the chain depends in part on how many efficient consumers exploit their prey [12]. The chain dynamic models of food webs are often unpredictable because different assumptions about mechanisms (e.g., co-limitation by predators and resources) lead to different predictions (e.g., consumer efficiency at a multi-trophic level) [13]. Among various processes that fit these premises, frugivory is an ideal candidate for these ecological and evolutionary analyses [14–16]. Patterns and processes of predation/dispersal of seeds are highly structured and coevolved spatiotemporally [14], with potential alterations promoted both by past human drivers [2] and current disturbances [9]. Plant-animal interactions within a community should consider the energy used at the individual level [14, 17].

Mammals and birds disperse about 90% of the woody plant species that produce fruits in Neotropical environments, generating an important feedback to plant fitness and forest regeneration [15, 18]. In the Atlantic Forest of South America, the family Myrtaceae is an important group of woody species and resource provider for a range of vertebrate taxa [19–20]. Myrtaceae species are the main components of lower altitude rainforests, where there can be over 50 species in a relatively small space (~1 ha) [21–22]. In subtropical Atlantic Forest, Myrtaceae in general are closely associated with the threatened conifer *Araucaria angustifolia* (Araucaria), which form Ombrophilous Mixed Forest (FOM) [23]. Forests dominated by Araucarias have been used as a proxy for conservation strategies [24]. Within FOM there is a vegetation mosaic of highlands (e.g., upper montane forest, swamps, and native grassland) that is spatially restricted and highly endangered mainly due to climate change [24–26]. In interspersed forest patches in this mosaic, Araucarias dominate the upper stratum, while Myrtaceae and Lauraceae are the main families that form the middle and lower strata [27–28].

A few Myrtaceae species dominate the flora in highlands, which are usually characterized by small fruits (< 1 cm of diameter) [29–30]. An exception to this is *Acca sellowiana* (Berg) Burret (Feijoa). Feijoa is a tree native to Southern Brazil and northern Uruguay that is restricted to areas in Southern Brazil over 800 meters above sea level (m.a.s.l.) associated with FOM [23, 31]. Feijoa is pollinated by birds and bees [32–33], and produces large fruits (20 to 250 g) with small seeds (~3 mm) [29]. Like Araucaria in local landscapes, Feijoa has been subjected to historical anthropogenic influence promoting a certain level of domestication of some populations *in situ*, which has resulted in increased fruit size and productivity by human selection [23, 34–36]. Currently, within FOM, Feijoa is used in several ways (circa 14) by local people that manage the species via pruning, transplanting, propagation by seeds and vegetative propagation [23]. Its domestication is incipient [23], a process that exhibits phenotypic variation within the range normally found in wild populations [37–39]. Moreover, the expansion of FOM by pre-Columbian people (i.e., indigenous Xokleng and Kaingang) favored the expansion of Feijoa and other associated Myrtaceae [23, 35]. In addition, the past and contemporaneous management of Feijoa and Araucaria increased the availability of resources in FOM [23, 35].

Plant and animal domestication is the most important development of human history since 13,000 years ago, and changed the course of humanity [40]. Since Darwin (1859), domesticated systems have played a critical role in the development and testing of evolutionary theory [41]. Management of native fruits *in situ*, both by indigenous populations and local farmers, is a recognized conservation practice that contributes to genetic diversity and several ecological processes through downstream propagation within biological networks [42–45]. Another outstanding characteristic of local landscape domestication—human intervention in the landscape components resulting in changes in evolution, ecology, interactions and species demography [3, 37]—is the presence of *Bos taurus* (cattle) and its impacts [36]. Cattle is an allochthone element that has been managed for a long time (> 100 years) by local small-scale landowners in highlands of subtropical Atlantic Forest [36]. Understanding the complex ecological legacy of management in past landscapes and their use components is important when debates are raging about the future of forests in relation to new human colonization dynamics, climate change, contemporaneous political conflict and defaunation [2, 46].

Currently, primary dispersion of Feijoa is barochoric [47], because the distribution region of Feijoa overlap areas under a constant defaunation of large-bodied vertebrates [8], remaining only a residual diversity reduced to a pale shadow of the once amazing biota of the Atlantic Forest. Yet, Feijoa still has a *posthoc* association with frugivores [29]. The functional loss of large fruit-eating vertebrates can decrease Feijoa fitness near conspecific adults [16, 48], disrupts the natural regeneration dynamics of plants, and changes the main components of the dispersal process [16, 49]. There is little empirical information on the interaction of Feijoa with its associated fauna, although there may be many species that feed on its fruits, as found for other Myrtaceae species, resulting in feedback that helps maintain ecosystem processes [13].

Therefore, as a temporary alternative during the austral summer-autumn with scarce resources (mainly the absence of *pinhão*), Feijoa can act as an attractor, contributing to the structure of animal communities and can maintain the residual diversity of vertebrates in Southern Brazil highlands. The aim of this study was to provide a scenario of the trophic relations between Feijoa (a species under incipient domestication and an alternative resource in threatened highlands) and local wildlife (which has experienced defaunation effects in a landscape influenced by historical management), to evaluate the space-time congruence between diversity patterns and resource productivity. Additionally, we aimed to evaluate the influence of the environment, the spatial distance among sites, the Feijoa domestication evidence, and the resource offer on the intensity of frugivory. We posed three hypotheses: (1) mammals and birds that remove fruits of *Acca sellowiana*–assuming *a priori* defaunated sites—are small- to medium-bodied frugivores-herbivore-omnivores and the cattle; (2) the seasonal Feijoa fruit structures the vertebrate fauna in space and time, increasing the diversity patterns in congruence with fruit offer. The productivity is linked with the incipient domestication process, and the positive effect of fruit offer include species that do not eat fruits, an implication of bottom-up control with historical influences; and (3) the number of interactions are related to environmental patterns, spatial distances, Feijoa domestication evidence and resource productivity. Both this factors directly or indirectly reflects local landscape domestication as a whole.

## Material and methods

### Ethics statement

Data collection was authorized based on license number 47255 from Instituto Chico Mendes de Conservação da Biodiversidade (ICMBio). Within private land (Reserva Particular do Patrimônio Natural (RPPNs)) we confirm that the owner of the land gave permission to conduct the study on this site. We confirm that the field studies did not involve manipulation of endangered or protected species, only species records via camera-trap. Vertebrate records were performed with non-invasive sampling (i.e. camera-trap). The work was not submitted to an Institutional Animal Care and Use Committee (IACUC) or equivalent animal ethics committee, because the data were collected only with camera-trap. Sampling procedures and/or experimental manipulations were reviewed or specifically approved as part of obtaining the field permit by the license number 47255 from Instituto Chico Mendes de Conservação da Biodiversidade (ICMBio).

### Research areas and design

**Fig 1:**
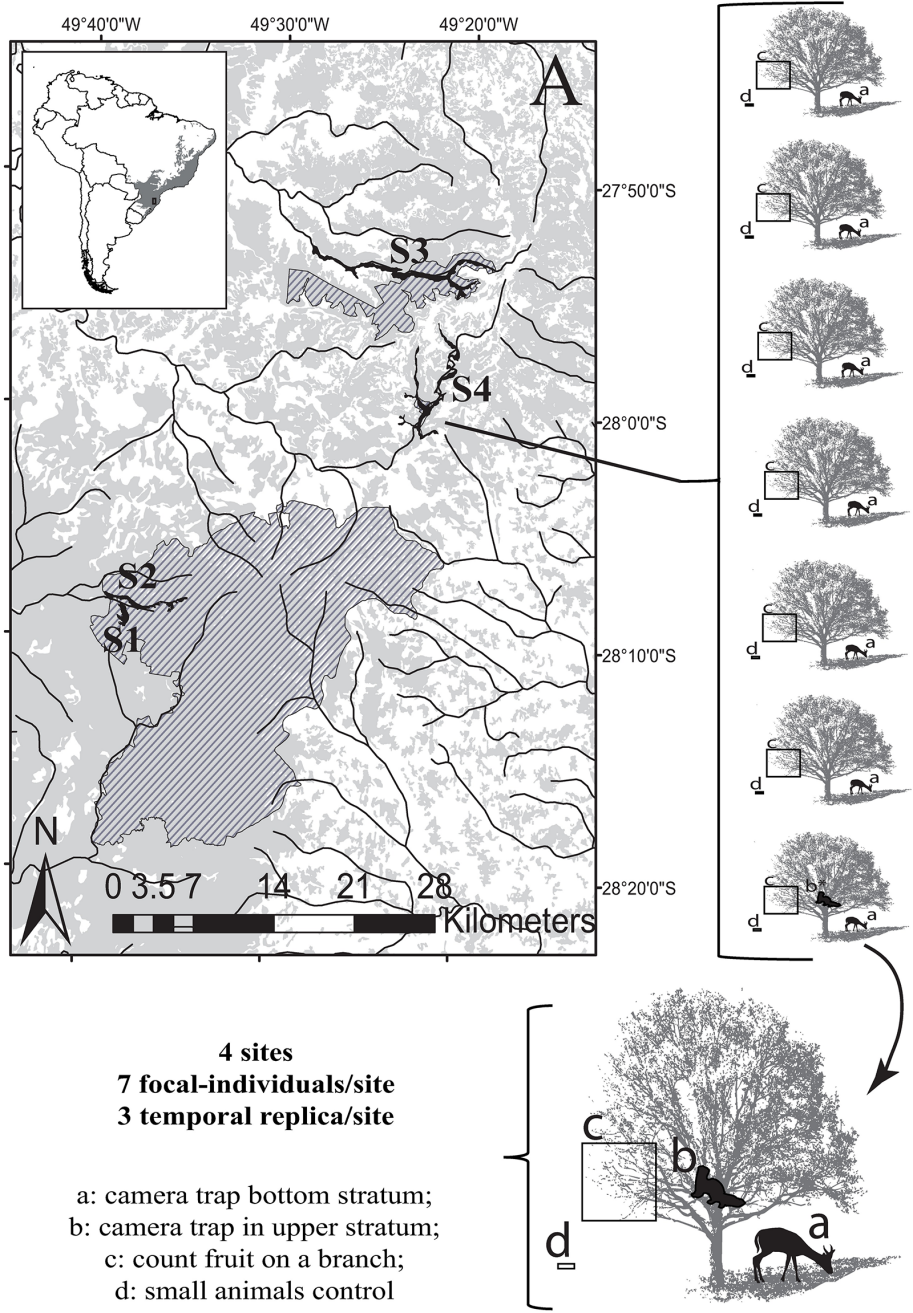
Research areas (political size dotted), sites and sample design to evaluate the removal of *Acca sellowiana* fruit by vertebrate fauna (mammals and birds) in highlands of subtropical Atlantic Forest, Brazil. In black: polygon of the site (based on lowland area or fragment size); in gray: the remnant of Atlantic Forest in years 2008–2010; lines in black: rivers. S1 and S2: São Joaquim National Park; S3: surrounding of RPPN Grande Floresta das Araucárias; and S4: RPPN Leão da Montanha.

We selected four sites in protected areas of subtropical Atlantic Forest highlands, in southern Brazil: within São Joaquim National Park (S1 and S2), surroundings of RPPN Grande Floresta das Araucárias (S3), and within RPPN Leão da Montanha (S4) (Fig 1; S1 Table). All areas fall within the humid subtropics (Cfa: Köppen-Geiger classification) [50], and are unequal in age and terms of protection, but share similarities in management and historical exploitation [36, 51]. Sites lie within a radius of circa 40 km and—based on previous studies and unpublished data—has a similar composition and richness of fauna, including defaunation indexes, and presumably a similar biota at pre-Columbian era [6; Bogoni et al. unpublished data]. The areas feature elements of cultural landscapes, which include anthropogenic forests with different intensities of past and present use [35–36] and regeneration stages, formed by Araucaria Forest (FOM), cloud forest, and native grassland [52]. These areas represent a mosaic of forest patches and high-altitude grassland composed by old rural proprieties destined for cattle breeding, logging and *Pinhão* extraction. These old proprieties were abandoned for more than 10 years, and are characterized for the presence of rock structures to retain the cattle (*taipas*), the foundation of old-houses (*taperas*), and other structures linked to past livestock activities. An exception is in S3 that remain with intensive livestock activity. Moreover, in the sites S1, S2 and S4 have the presence of Feijoa trees typically maintained with management (e.g., traces of pruning, producing largest fruits and located near to old proprieties foundations) [23, 37]. These changes in species traits starting from wild ancestors (e.g., changes on the morphology of aerial vegetative parts and selection of fruits size and quality) are classical syndromes of domestication in food crops [53].

At each site, due to the supply of equipment, we chose seven focal individuals of Feijoa based on fruit presence and, as a secondary factor, the distance among them larger than 150 m. For each focal individual, we recorded the fauna in the lower stratum (~30 cm from the ground) using a camera trap (*Bushnell*: Trophy Cam HD, Model: 119537c, Bushnell Outdoor Products Canada, Ontario, Canada), including three temporal replicas during 2015–2016 (autumn in the beginning of 2015 (TR1), middle of spring of 2015 (without fruits:TR2) and autumn in the beginning of 2016 (TR3)). For the focal individuals 1, 4 and 7 of each site, we installed camera traps at ≥ 1.7 m high (upper stratum) and attached five fruits on each tree with nails. Since is technically impossible attach fruit in locations that they would occur naturally (e.g., tips of branches), Feijoa fruits were attached in a trunk capable of receiving the small nails (12 x 12 mm) and that it was possible to install the camera trap immediately ahead of these attached fruits. Due to the number of available camera traps, in TR2 and TR3 we only used one camera trap in the upper stratum that was installed on focal individual one. This reduction in camera traps within upper stratum was chosen because according to the TR1 the main frugivory activity was performed on lower stratum. Thus, we can keep the equal number of focal individual (seven) in each temporal replica.

We left the camera traps active in each site for at least 30 days or for 60 days in sites where available fruits exceeded 30 days; in these cases, after 30 days we inspected the equipment. Thus, a total of 40 (or 32) camera traps were used, programmed to record footage for 45 seconds, with an interval of 45 seconds between recordings, totaling 4,470 traps/day (Fig 1). In both strata and temporal replica, we analyzed the independence of interactions according to fruits consumed, with sequential videos of the same species, where consuming the same fruit was considered a single interaction record. We established the spectrum of fruit removal by the following criteria: (1) sinzoochory, the quantity of whole fruits taken and transported away from the location; and (2) endozoochory, fruits consumed locally [47]. Additionally, we counted fruit removal by small animals at each focal individual in TR1, using 3-inch pipes stuck to the ground, which contained 5 Feijoa fruits (prohibiting the access of medium to large animals) (Fig 1).

### Resource availability, environmental and spatial data

For each temporal replica with fruits (TR1 and TR3), at the time of camera trap installation (t0), we counted the number of fruits available on the ground below the canopy of the focal individual. We estimated the productivity of every tree by the number fruits on the main branch (or entire tree when possible) multiplied by the number of equal-sized branches on the focal individual (modified from Clark et al. 2005) [54]. At intervals of 30 days (t1) and 60 days (t2), we returned to count the amount of fruit on the tree and quantified fruit availability during camera trapping. In t0, t1 and t2, we weighed five fruits from each individual with a manual precision balance, and calculated the biomass for each time. Furthermore, in TR1 we: (1) calculated the fruit decomposition time using 20 fruits chosen randomly at a single moment, left under conditions similar to the sites; and (2) counted the number of seeds in 15 fruits.

In the first year of the study, we recorded the focal-individual canopy area and the distance of the focal individual to the closest Feijoa adult (with reproductive signals or circumference at breast height (CBH) ≥ 15 cm) [55]. To characterize the site habitats, we measured environmental features using an adapted point quadrant (S1 Fig) method [56]. For each focal individual, we measured the canopy cover percentage (from photographs taken 1.5 m above the ground, at four points (N, S, E and W) five meters from each individual), diameter, height and distance to the nearest tree and shrub. We also estimated the percentage of ground cover (litterfall and herbaceous green cover within 1 m^2^ delimited on the ground), slope, orientation, intensity of use of the area by livestock (ordinal: 0 (absence) to 4 (high)) and actual human presence (binary: 0 (low) and 1 (moderate)).

We also noted the presence of evidences of management in the focal individuals of Feijoa (e.g., traces of pruning, tree and fruit size, fruit variety (e.g., thin bark, more appreciated for local people consumes) and proximity of places with past human activities), that can indicate the continuity of domestication process [23, 37, 53] (S1 Fig). We supported this aforementioned information with semi-structured questionaries’ applied to local people [Bogoni et al. unpublished data]. Thus, we obtained a proportion of domesticated Feijoas within sites based on qualitative data. For this, we considered presence Feijoas with management evidence (domesticated Feijoas) and absent for individuals of Feijoa (“under-domesticated” Feijoas) with inconspicuous domestication evidence, although it may present at a lower level (e.g., molecular)) [37, 53].

Moreover, we delimited one plot of 1,600 m^2^ in each site to determine the demographic characteristics (account of adults) of Feijoa within the sites (c). We measured landscape characteristics of the sites and the distance between focal individuals and sites through georeferenced data. Using GPS, satellite images and QGis software [57], we measured the linear distance from the focal individual to the closest watercourse, to the nearest open area and to the nearest fragment of native forest [58]. Additionally, we obtained the size (as a polygon) of the forest fragment or lowland (i.e., *várzea* or flat areas of river banks, an environment where Feijoa often occurs) based on *in situ* personal observations and Santos et al. (2009) [23].

### Data analysis

We performed a principal component analysis (PCA) of correlation to evaluate the ordination sampling sites across environmental and landscape parameters, including the proportion of managed Feijoa trees within sites [59–60]. Descriptively, we explored the spatial distance, demography, and fruit productivity of Feijoas per site and temporal replicates (TR1 and TR3), using central tendency and variation measures. We analyzed the structure of the communities using diversity descriptors (i.e., species richness and rarefaction curves with confidence intervals of 95% for each replica) [61–62]. For each site, we only used independent video recordings, excluding records of the same species at the same focal individual for time intervals shorter than 1 hour [63], adjusted by site sampling effort, and assuming spatial dependence among focal individuals for mammals and birds.

We analyzed the topology of weighted two-mode frugivory networks per site for each temporal replicate and per focal individual [64–65], in which a set of nodes representing frugivores species is connected to another set of nodes representing sampling sites or focal individual where they were recorded consuming Feijoa fruits. We calculated one quantitative metric: (a) modularity, and three qualitative (binary) metrics: (b) average degree (c) connectance and (d) nestedness [66–67]. Degree is the number of interactions each node has. Modularity (M) quantifies the tendency of the nodes to cluster into cohesive groups that are more connected among themselves than with the rest of the network [66]. Connectance is the proportion of realized links, that is the ratio between the number of interactions observed in relation to the possible interactions [67]. Nestedness (N) measures the degree by which the nested network, showing a possible hierarchical pattern [68]. In case of nestedness, one or more sites can be a subset of other sites with largest species richness or largest interaction number. For modularity, we used Newman’s metric [64] and compared its empirical value with a benchmark distribution of modularity values calculated to an ensemble of 1000 theoretical matrices created by a null model, in which species degree ranges between zero and the mean of the degree of the real network. Significance (p ≤ 0.05) was based on the ratio at which larger or equal to the observed M value occurred in the null model [69]. For nestedness, we used the NODF metric [68]. NODF ranges from zero, when the matrix is perfectly non-nested, to 100, when the matrix is perfectly nested [70]. We also compared the NODF value of empirical network with a benchmark distribution generated by 1000 theoretical matrices generated by a null model based on a probability matrix (null model 2 of Bascompte et al. 2003) [69] and adopting the same aforementioned criterion for M significance [69]. Additionally, to show resource sharing graphically, we build the network with the deconstructed interactions per temporal replica in: (1) disperser groups (mammals and birds); (2) dispersal syndromes (endozoochory and sinzoochory); (3) vertical strata (upper and lower); (4) time (t0 to t1 and t1 to t2); and (5) circadian condition (day and night).

We performed a variation partitioning analysis to assess the environmental (including Feijoa domestication evidence), spatial distance and productivity variation effects on interactions [59–60] at the focal individual scale, considering the two groups (native mammals and birds) separated. However, *a priori*, we conducted a principal component analysis of neighboring arrays (PCNM) to determine significant spatial components obtained by the array of distance between focal individuals. The PCNMs represent the spatial relationship between sampling sites and can be used as predictors of variation. Only positive PCNMs were selected according to spatial autocorrelation obtained by Moran’s index [59–60].

To partition the variation, we used the forward selection method (to reduce the likelihood of a type I error) [71] to select a subset of significant predictor variables based on the non-multicollinear data (tested *a priori* via the variance inflation factors) [72]. Variation partitioning performs RDAs for all predictors, and individually for each set of predictors, removing effects of other predictors (e.g., RDA for the environment removes effects of spatial distance). The proportions explained by predictors were represented by Ezekiel-adjusted R^2^ [73]. For the variation partitioning, we corrected the asymmetry in the interaction number using Hellinger transformation. The asymmetry of the environment and landscape features (numerical magnitudes) was transformed by standardization, and the resource availability (total biomass) was logarithmized [60]. We based the significance of variation partitioning tests (p ≤ 0.05) on 999 permutations. For other analyses, used the original, untransformed datasets. We performed all analyses in R [74] with the sna [75], bipartite [76], vegan [77] and Packfor [78] packages.

## Results

### Environment, spatial distances and demographic features

Environment and landscape features, analyzed via PCA, explained 85.4% of total data variation among sites, with 52.7% of the explanation related to axis one (linked mainly to slope and lowland/fragment size) and 32.7% of explanation related to axis two (linked to tree height and distance to nearest fragment). The sites are distinct in the following aspects: S1 is the furthest from a watercourse (340 m); S2 has higher adult density (each one by 5.5 m apart); S3, besides the intense presence of cattle, is characterized by the greater area (697 ha) of “*várzea*”; and S4 is the furthest from an open area (105 m) (S2 Fig). Although not the main factor in the ordination analysis, at different intensities or replications, there was a real or virtual presence of cattle in all sites. The average distance between sites is 23.7 km (±11.3; 1.6 to 34.3 km). The distance among focal individuals is 261.5 m (±182.4; 11.3 to 496.5 m) in S1, 446.7 m (±320.9; 11.2 to 1000 m) in S2, 346.4 m (±246.7; 7.7 to 716 m) in S3, and 584.9 m (±356.4; 88 to 1167 m) in S4. Considering all sites, and based on the distance among Feijoa trees and demographic plots, there is an average of 48.8 (±39.8) Feijoa adults per hectare. The proportion of Feijoa trees (focal individuals) with clear evidence of past management (i.e. domestication) was 85.7% (6 in 7) in S1, 57% (4 in 7) in S2 and S4 and 0% in S3 (Table 1).

**Table 1:** Density, domestication evidence, and productivity of *Acca sellowiana* at four sites and two temporal replicas with fruits in subtropical Atlantic Forest highlands, Brazil. S1 and S2: São Joaquim National Park; S3: surrounding of RPPN Grande Floresta das Araucárias; and S4: RPPN Leão da Montanha; A: adults.

Replica		Density	Domestication evidence	Fruits		Biomass		
Spatial	Temporal	Ind/ha (A)	N in 7 focal individuals (%)	N total/Site (±SD)	N total/ha (±SD)	Average (g/fruit (±SD))	Kg/Site	Kg/ha
S1	TR1	**-**		2,661	14,255.3	46.2 (16.5)	123.7	662.7
	TR3	**-**		924	4,950	24.2 (12.1)	22.4	120.0
	**Mean**	**37.5**	**6/7 (85.7%)**	**1,792.5 (1,228.2)**	**9,602.7 (6,580)**	**35.2 (15.6)**	**73.1 (71.3)**	**391.4 (383)**
S2	TR1	**-**		844	1,700.1	30.6 (7.9)	25.8	52.0
	TR3	**-**		72	145.1	27.4 (15.1)	1.9	3.8
	**Mean**	**14.1**	**4/7 (57%)**	**458 (545.9)**	**922.6 (1,100)**	**29.0 (2.3)**	**13.9 (16.9)**	**27.9 (34)**
S3	TR1	**-**		313	4,753.2	28.1 (14.4)	8.8	133.6
	TR3	**-**		96	1,457.8	12.8 (6.2)	1.2	18.2
	**Mean**	**106.3**	**0/7 (0%)**	**204.5 (153.4)**	**3,105.5 (2,330)**	**20.5 (10.8)**	**5.0 (5.4)**	**75.9 (82)**
S4	TR1	**-**		696	3728.6	39.8 (15.8)	34.8	186.4
	TR3	**-**		369	1976.8	27.2 (11.9)	10.0	53.6
	**Mean**	**37.5**	**4/7 (57%)**	**532.5 (231.2)**	**2,852.7 (1,239)**	**33.5 (9.0)**	**22.4 (17.5)**	**120.0 (94)**
**Average**		**48.8 (39.8)**	**50 (36)**	**746.9 (838.1)**	**4,120.9 (3,782.4)**	**29.6 (10.0)**	**28.6 (40.2)**	**153.8 (162.8)**

### Productivity and interactions

Spatially, the productivity among sites varied from dozens to more than 2,500 fruits (average 746.9; SD ±838.1; totaling 5,975 fruits), and the biomass was also highly variable (3.03 ±5.98 kg per individual; 28.6 ±40.2 per site) (S3 Fig). These values, extrapolated for the density of adults per hectare, maintained high spatial variation (4,120.9 ±3782.4 fruits/ha^-1^ and 153.8 ±162.8 kg/ha^-1^) with major productivity in S1 and S4. Temporally, we also found high variation in the number of fruits and biomass, with a 67.6% decrease in fruit availability between TR3 with TR1 (Table 1). The fruits rotted in nine (±2.4) days and the average number of seeds per fruit was 61.9 (±36.4).

**Fig 2:**
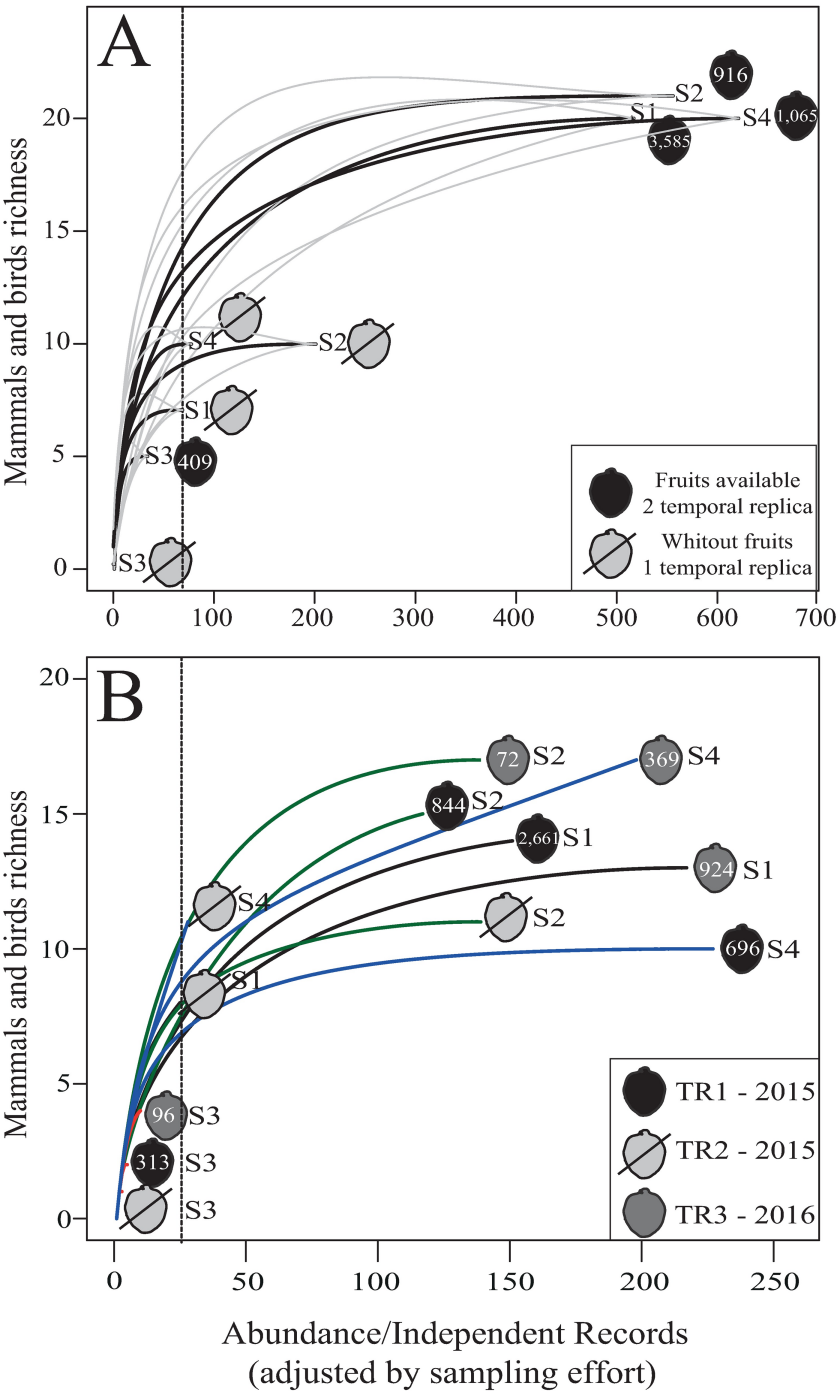
(A) Rarefaction curve (with a confidence interval of 95% in gray) for all independent records (considering two replicas with fruit and one without fruits) of mammal and bird species (except exotic) during evaluation of fruit removal from *Acca sellowiana* in four sites in subtropical Atlantic Forest highlands, Brazil; (B) Rarefaction by spatial and temporal replicas for all independent records of mammals and birds species (except exotic species) during evaluation of fruit removal from *Acca sellowiana* in four sites in subtropical Atlantic Forest highlands, Brazil. Independent records were adjusted by sampling effort (camera-trap/day/replica). S1 and S2: São Joaquim National Park; S3: surrounding of RPPN Grande Floresta das Araucárias; and S4: RPPN Leão da Montanha.

Camera traps recorded a total of 1,948 videos (1,461 minutes), including 470 independent interactions and 1,141 independent records according to both our criteria, with 37 native species recorded when considering Cricetidae as a unique species (S2 Table). Rarefaction curves considering all the native species recorded (independently of interaction with Feijoa) showed there was a significant temporal difference in species richness, i.e., between presence (TR1 and TR3) and absence (TR2) of the resource when comparing a minimum of independent records (S1: 72; disregarding S3 due low richness). However, among sites, there was no significant difference in richness, with confidence intervals overlapping, except for S3 (Fig 2). This pattern was maintained when the temporal replicas were separate (Fig 2; S4 Fig).

**Fig 3:**
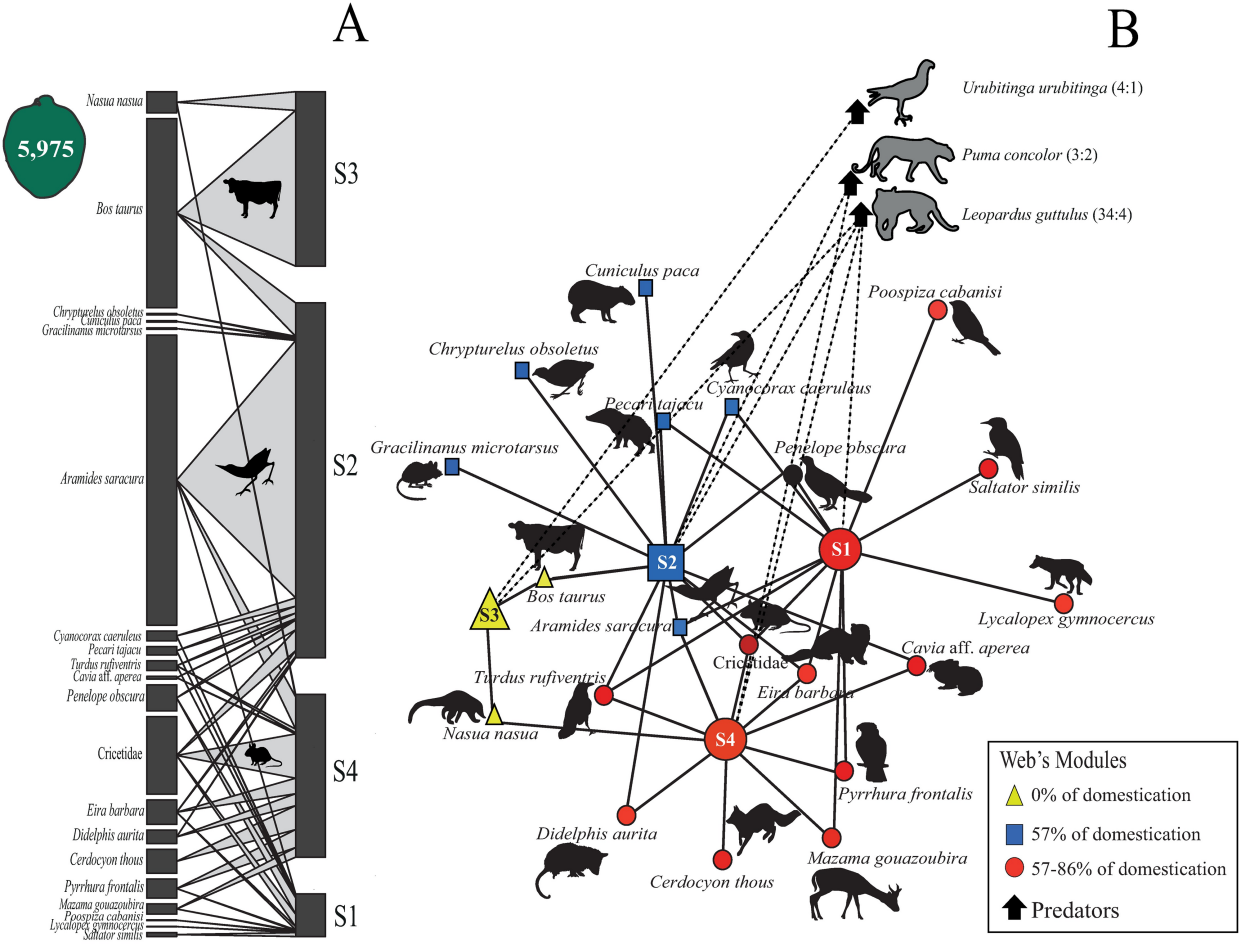
Topology of the total network by the site for years 2015 and 2016 to evaluate the removal of *Acca sellowiana* fruit by vertebrate fauna (mammals and birds) in the subtropical Atlantic Forest highlands, Brazil. (A) Two-mode network (bipartite) network considering two years of research; and (B) Two-mode network showing the modularity of different groups. S1 and S2: São Joaquim National Park; S3: surrounding of RPPN Grande Floresta das Araucárias; and S4: RPPN Leão da Montanha. Figure in high resolution, for details, apply zoom. In (A) the left column and their thickness represents the species that performed frugivory and the frugivory intensity (species degree); the right column represent and their thickness represents the sites and the intensity of frugivory in each one; transverse lines represent the links between species and sites. In (B) each minor point represents the species and larger points represents the sites; the lines linking the nodes (species and sites) represents the interaction among them; dotted links represent the presence of each predator in each site.

During the two years of sampling, 20 species (including cattle and Cricetidae: S5 Fig and S1 Movie File) interacted with Feijoa, with an average sites degree of 14.41 (±10.1), a Feijoa fruit removal of 9.6% representing 572 fruits removed and more than 35,400 seeds. Among sites, when both temporal replicas were combined the removal proportion were 3.35% for S1, 27.84% for S2, 13.70% for S3 and 12.77% for S4. In TR1, the average removal was 14.39% (3.2% to 28.1% among sites, with the same pattern as the total average) made by 19 species: 2.83% by 10 native mammals (including Cricetidae), 7.28% by eight native birds, and 4.29% by cattle. In TR3, the average removal was 13.41% (3.57 to 25.0% among sites) made by 13 species: 7.80% by eight native mammals, 2.75% by four native birds and 2.86% by cattle (Fig 3). Among native mammals, Cricetidae (species degree of 6.09), *Eira barbara* (1.91), *Cerdocyon thous* (1.88), *Nasua nasua* (1.66) and *Didelphis aurita* (1.05) are the main species that remove fruits. *Aramides saracura* (22.76), *Penelope obscura* (2.05), *Pyrrhura frontalis* (1.50), *Turdus rufiventris* (0.78) and *Cyanocorax caeruleus* (0.77) is the main bird species that remove fruits (Fig 3; S6 Fig).

Total connectance of the network was high, with 0.46 for two years, 0.41 for TR1 and 0.38 for TR3. Total modularity of the network was statistically higher than null expectancy (M^Total^_obs_ = 0.42; M^Total^_null_ = 0.14; p < 0.01) and identified three different modules containing S1 and S4, S2 alone, and S3 alone (Fig 3). In TR1, modularity (M^TR1^_obs_ = 0.36; M ^TR1^_null_ = 0.14; p < 0.01) identified two modules, and in TR3 each site was identified as one module and the modularity was significantly higher than null expectancy (M ^TR3^_obs_ = 0.45; M ^TR3^_null_ = 0.16; p < 0.01). The total spatial nestedness was intermediary and did not differ from the null expectancy (N^Total^_obs_ = 51.4; N^Total^_null_ = 47.3; p = 0.19), as in TR1 and TR3 (N ^TR1^_obs_ = 46.7; N ^TR1^_null_ = 40.6; p = 0.08; N ^TR3^_obs_ = 35.7; N ^TR3^_null_ = 39.0; p = 0.26). The network, in all sites and temporal replicas, showed higher variations in removal types, vertical stratum, fruit duration and circadian period (S7 Fig). The removal of small animals (including invertebrates) using three-inch control pipes was 31.56%. Additionally, we made 49 independent records of medium- to large-bodied carnivores (*Puma concolor*, *Leopardus guttulus*, *Leopardus wiedii*, *Parabuteo leucorrhous* and *Urubitinga urubitinga*), 79.6% (39) of each during replicas with fruits (TR1 and TR3) (Fig 3).

**Fig 4:**
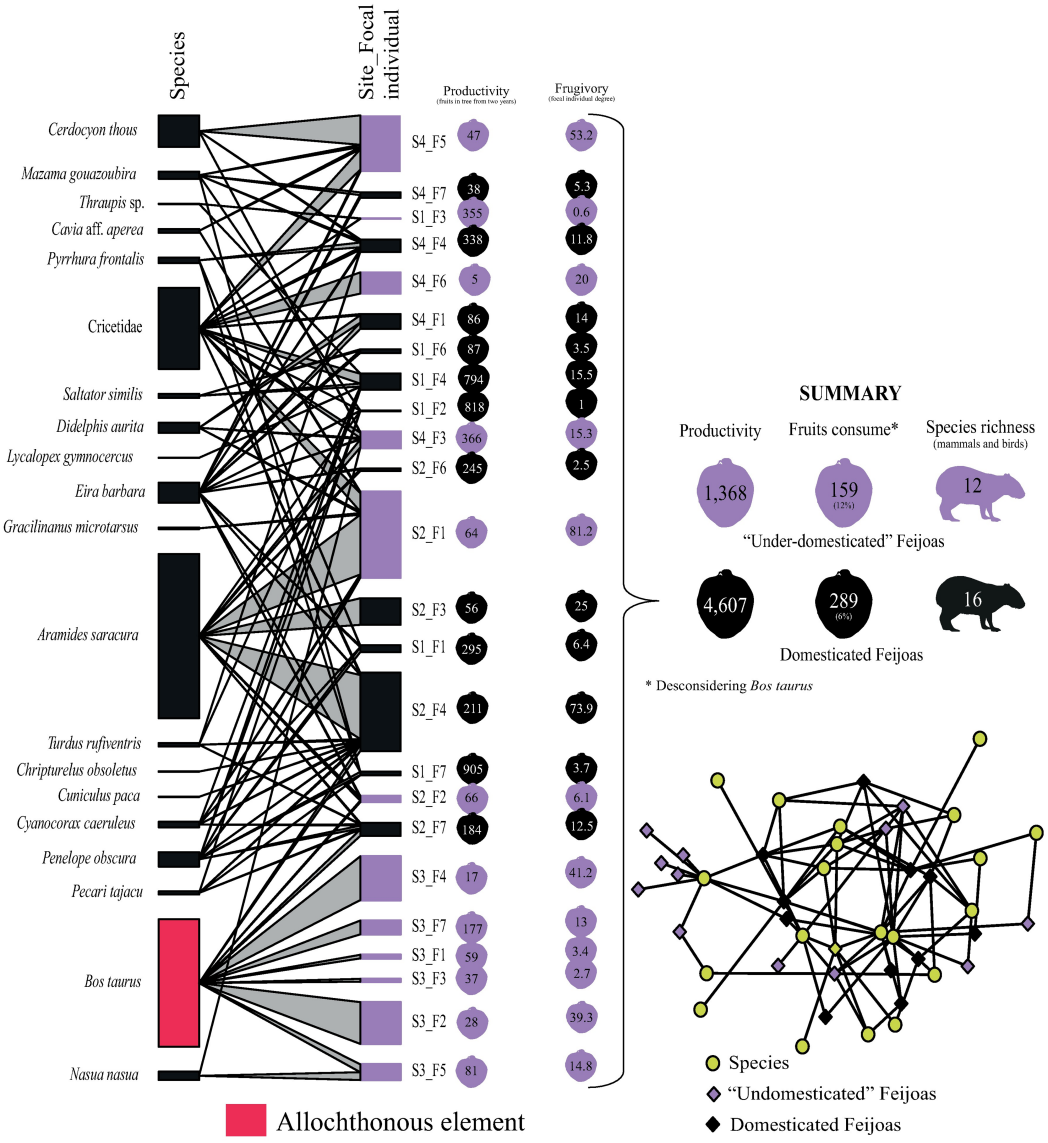
Topology of the total network by focal individual (Feijoas trees) for years 2015 and 2016 to evaluate the removal of *Acca sellowiana* fruit by vertebrate fauna (mammals and birds) in the subtropical Atlantic Forest highlands, Brazil. Bipartite network considering two years of research. S1 and S2: São Joaquim National Park; S3: surrounding of RPPN Grande Floresta das Araucárias; and S4: RPPN Leão da Montanha. Figure in high resolution, for details, apply zoom. The graphic representation is the same contained in the Fig 3.

Network analysis per focal individual jointing all temporal replica with Feijoa fruit presence sowed a focal individual degree of 16.6 (±21.8). Considering Feijoas with signals of domestication the focal individual degree was 10.8 (±19) while Feijoas without domestication evidence (“under-domesticated”) was a degree of 22.6 (±23.6). Whereas Feijoas domesticated that has interaction with vertebrates summed 4,607 fruits produced (329.1 (±292.3)) fruits per tree representing circa four times more than “under-domesticated” Feijoas) and the frugivory was ~45% larger than in “under-domesticated” Feijoas. Moreover, among the Feijoas domesticated 16 species (mammals and birds) performed frugivory, representing four more species than in “under-domesticated” Feijoas (Fig 4). In this condition, the network connectance was 0.15, the modularity and nestedness of were statistically higher than null expectancy (M^Focal_ind^_obs_ = 0.12; M^Focal_ind^_null_ = 0.07; p < 0.01; and N^Focal_ind^_obs_ = 27.8; N^Focal_ind^_null_ = 20.9; p < 0.01). The main species that performed frugivory at focal individual scale were the same observed when the data was jointed at site scale (e.g., *Aramides saracura*, *Bos taurus*, Cricetidae, *Cerdocyon thous*, *Eira barbara*, *Penelope obscura* and *Didelphis aurita*) (Fig 4).

### Trends in influence of environment, spatial distance, temporal replicas and resource availability on frugivory

**Fig 5:**
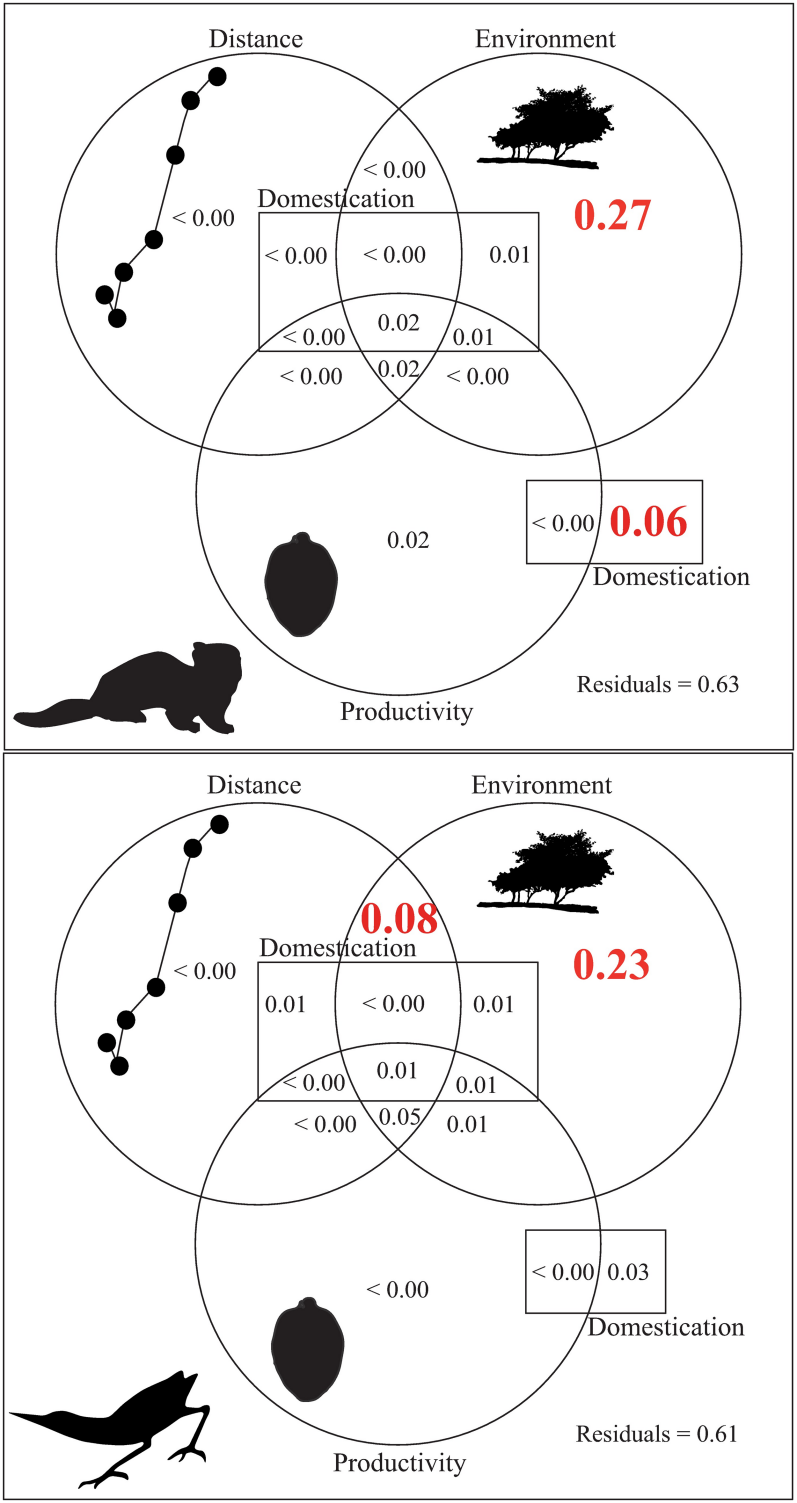
Variation partitioning by focal individuals of *Acca sellowiana* and their interaction with vertebrate fauna (mammals (above) and birds (below)) in four sites in subtropical Atlantic Forest highlands, Brazil. The value in red was statistically significant at p ≤ 0.05 based on 999 permutations.

Numerically, the mammal and bird interactions for different focal individuals in each site are influenced only by habitat structure, represented by canopy area, percentage of herbaceous coverage, fragment/lowland area and distance to water body/drain trench (only for birds). Thus, the variation partitioning analysis for all interactions over two years showed that the habitat explained 27% (r^2^adj = 0.27; F = 4.39; p < 0.01) of the interaction variation of mammals and 23% (r^2^adj = 0.23; F = 3.08; p < 0.01) of the interactions of birds with Feijoa fruits (Fig 4). Moreover, the proportion of domesticated trees within the sites were responsible for the 6% of variation of interaction between Feijoa fruits and mammals (r^2^adj = 0.06; F = 2.96; p = 0.03). For birds, an additional explanation (8%) was attributed to the combination of environment and distance among focal individual (r^2^adj = 0.08; F = 3.01; p = 0.02) (Fig 5).

## Discussion

The identification of the selective forces on plant dispersal engenders theoretical and empirical issues [15]. The game of seed and fruit removal is played by mobile predators in search of sessile prey; discharge by a single dispersal move, seed chemistry, parental morphology and behavior, evolutionary change [14] and this game are influenced by human actions [2, 16]. Accordingly, our main findings show that Feijoa acts as an attractor of fauna and so contributes to the community structuring of mammals and birds. The diversity of fauna had a seasonal diversity increase—especially in terms of number of independent records—in congruence with spatiotemporal fruit offer, and the incipient domestication of Feijoa continues to favor the associated fauna, with a maintenance of local residual diversity, supplying food during the seasonal absence of *pinhão*. For example, a recent study in the Atlantic Forest of South America performed in 118 areas at a neutral perspective—generally without the presence of Feijoa—showed an alpha diversity of medium- to large-bodied mammalian of 9.86 species [79]. This average is numerically lesser than the richness recorded in this study (i.e. 12 medium- to large-bodied mammals species within a stressful environment due to the high altitude). An ambiguity of several contingency and determinism processes are able to determine the diversity pattern in a specific place [79]. Yet, the pattern showed in our result may be directly related with the seasonal resources of FOM (e.g., Feijoa and *pinhão* in alternation) that are able to maintain the residual richness of species that once was larger and nowadays range from nine to 16 species of medium- to large-bodied mammals [8]. Moreover, the process of landscape domestication, containing managed trees in anthropogenic forests, transcends the limits of natural distribution and abundance patterns, causing the dynamic of species interactions (e.g., seed removal) to be influenced by accumulated past and present human actions [2].

Whereby Feijoa domestication evidence—that was performed via management of trees near to local people houses [23, 53]—observed in this study was responsible for the 6% of the variation in interactions between mammals and Feijoa fruits. Besides, the sites with more Feijoa trees under these aforementioned conditions (e.g., within S1) showed the largest diversity of vertebrates, presumably a cascade-effect of past human activities. At focal individual scale, this relationship also was highlighted because: (1) the fruit removal on Feijoa domesticated was circa 45% more than observed in Feijoas without evidence of domestication; (2) no Feijoa tree had 100% of fruit removal, suggesting that the removal in both conditions could be larger than observed; and (3) the number of species that performed frugivory in domesticated Feijoas was 25% larger than in “under-domesticated” trees. Furthermore, domestication activities ensure the genetic diversity of Feijoa that is maintained *in situ* (native forests) and/or on farms [38, 53]. Feijoa presents a low effect of endogamy, large genetic divergence among the populations and high incidence of rare and exclusive alleles among some populations within the highlands (including our sites S1 and S2) of subtropical Atlantic Forest [39], a strong indicative of the anthropic activities of management on species and local landscapes [23, 38, 39, 53].

Excluding *Homo sapiens* and their past influences on forest composition [3, 35], information about dispersal agents of Brazilian Myrtaceae and the success of plant establishment after the dispersal process remains unknown [29]. The fauna ecologically associated with Feijoa can enhance the fitness and natural regeneration of this tree, as for other plants [14–16], ensuring the maintenance of forest diversity [79]. How groups of frugivores disperse plant seeds needs to be studied by evaluating: (1) the speed and the percentage of germination; (2) seed deposition pattern, influenced by the number of seeds and species combination in the deposition; and (3) plant establishment success within the community [80]. Thus, for example, if only 0.5% of the seeds removed (a very low number compared to other Myrtaceae that pass through animal guts (e.g., [81–84]) successfully germinate; we predict that Feijoa frugivory can generate ~178 new Feijoa seedling in two years. For animals, mainly because of more than 18 native species consume fruits, the Feijoa is an alternative resource during the season when food is scarce in highland areas that can contribute to maintaining diversity and populations [85]. Feijoa promoted well-structured trophic relationships [13, 86], due to the spatiotemporal increase in the quantity of medium- to large-bodied predators, congruent with fruit availability (e.g., [87]), an example of bottom-up forces in community structuring [13].

The array of frugivores can influence productivity, fruit and seed size, and demographic variations from both short- and long-term evolutionary scales, because evolutionary changes are underway for some plant species at sites without large fruit-eating species [16]. In the short-term, the productivity of Feijoa is related to pollination, dependent directly on birds and bees and indirectly on the environment and climatic variables [88]. Thus, in the long-term the sites can be subject to evolutionary changes in interaction patterns (e.g., reduction in fruit size) due to the absence of large native fruit-eating species (e.g., [16]. Therefore, decompensation is expected due to incipient domestication efforts aimed at increasing fruit size, quality and productivity of Feijoa [23]. However, Feijoa may be maintained by the presence of cattle in sites where they recently occurred, another remarkable human impact in a landscape [36] that favors large Feijoa fruits. Cattle presence and biomass were determinant factors for fruit removal, as well as the displacement of native fauna, evidenced by the low native richness in the site (S3) where the presence of cattle was outstanding. The contribution of cattle to Feijoa fruit removal was high, as found in other studies for seed and fruit removal in the presence of cattle [89–90], which can compromise seed viability [91]. Human activities can favor Feijoa fitness, and although past management maintained the diversity of interacting species that promoted propagation throughout the entire local network [43–44], some activities also paradoxically displaced the native fauna, especially livestock farming at large scales [46].

Many areas of Atlantic Forest have suffered from defaunation [8, 92–93]. The absence of animals that mainly remove seeds in all of the sites (*Tapirus terrestris* and *Tayassu pecari*: [94–95]) can promote an increase in seed predation, due high interactions with small rodents [95]. Our results agree with this pattern, since the Cricetidae was an important removal group that has the potential to significantly compromise the seed dispersal network [18, 93]. The connectance of the fruit-removal network was intermediary, indicating that Feijoa can drive interactions with the fauna in the sites and that it is partially compromised by defaunation (e.g., [93]). Modularity was higher than expected indicating weak groups of sites linked by some species in common (e.g., Cricetidae, *Aramides saracura* and *Eira barbara*), but with modules (e.g., S1 and S4, the most productive sites) internally stronger with a large variation between temporal replicas, showing an unstable dynamic in the removal of Feijoa fruits. Modularity increased with the link specificity, being stronger in insect herbivory networks than in pollination and seed-dispersal networks, characterized by low interaction specificity [66, 96]. Yet, the nestedness was intermediate and not statistically different than expected by chance. This reflects in the degree animals influence on the network [97] and is congruent with the connectance pattern of the network.

Among the main species in the networks, mammals with great plasticity and tolerance to environmental changes were notable (e.g., *Eira barbara*, *Cerdocyon thous*, *Nasua nasua* and *Didelphis aurita*). Studies about the diets of these mammals showed that home range and a large spectrum of food items, including many fruits (e.g., [98–102]), can result in spreading seeds from 3 km^2^ to 20 km^2^ [103]. Among birds, the main species have the largest body size in the sites, supposedly have a variable diet of food items, including fruits [104–105], and can disperse seeds over long distances [106–107]; although, there is a decrease in the number of seeds as the distance increases [108]. However, due to high variability in local relief and the restriction on the establishment and productivity of Feijoa at altitudes lower than 800 meters [23], the potential dispersal home range can be further reduced, which confirms the importance of conservation efforts to preserve the highlands and their environmental components. These environmental characteristics of the sites exerted significant influence on the interaction number at the focal-individual scale (based on variation partitioning). The relationship between species distribution and environmental characteristics is a major goal of ecology, and such relationships are likely to be important predictors of community structure [73].

The network partitioning showed a tendency of resource sharing and functional redundancy among species [109], not only for taxonomic groups but also in removal syndromes, vertical stratum, beginning and end of fruiting and activity time (day and night). Sites spatially closer to each other can be linked by Feijoa frugivores over the short- and medium-term, mainly because the distance is not significantly important to the amount of fruit removal and the main interactors can disperse seeds between sites over the short-term. Among sessile organisms, with seeds that represent the predominant mobile stage, dispersal is the primary spatial demographic process [110]. Yet, it also serves as a template for subsequent processes, such as predation, competition and mating [110], and evaluations of habitat loss, disturbances and landscape connectivity [93, 111].

Overall the patterns we found could be present in other fruit-eating and seed dispersal networks in different ecosystems, especially in Neotropical regions where the gaps are even greater [93, 112]. Thus, we can conclude that our hypotheses were totally or partially corroborated because Feijoa was ecologically related to small- to medium-bodied herbivore-omnivores, was the resource structuring the vertebrate fauna in space and time, was increasing the diversity patterns seasonally in congruence with the fruit offer, and the ecological relationships were related to environmental characteristics and Feijoa domestication (for mammals). Sequential years with lower productivity of Feijoa probably not would cause a local extinction, but can lead to a decrease in vertebrate population sizes. Long-term experimental studies can be developed to corroborate if this seasonal increase in diversity is propagating at long-term, reinforcing the importance of Feijoa in an environment with low productivity and high seasonality. Our insights allowed us to evaluate the magnitude of interactions between vertebrates and an incipient domesticated tree, in a cultural landscape and highly threatened environment (Atlantic Forest highlands), under a basal foodweb approach with implications for bottom-up and top-down forces. We contribute to understanding the relationship between mammals, birds, and fruit trees with concepts and results that can be replicated for other relationships between a sessile prey and mobile predators in any region or habitat under different gradients of human actions, environmental management, and historical ecology. This study shows how human actions can change not only the distribution patterns and abundance of species, but also the diversity and direction of interspecific interactions. The human footprint on forest composition can modify the ecological components of a landscape in a continuous cascading effect.

## Supporting information

S1 Table Characteristic of research areas and sampling sites used to evaluate the removal of *Acca sellowiana* fruit by vertebrate fauna (mammals and birds) in the subtropical Atlantic Forest highlands, Brazil. PSA: São Joaquim National Park; RGF: Reserva Particular do Patrimônio Natural (RPPN) Grande Floresta das Araucárias; RLM: RPPN Leão da Montanha; FOM: Mixed Ombrophilous Forest; UM: upper montane; AL: alluvial; m.a.s.l.: meters above sea level; SE: sampling effort (days); TR1: Temporal replica one (2015); TR2: Temporal replica two (2015, without fruit); TR3: Temporal replica three (2016).
(XLSX)

S2 Table Birds, mammals and exotic species recorded into four sampling sites used to evaluate the removal of *Acca sellowiana* fruit by vertebrates in the subtropical Atlantic Forest highlands, Brazil. (XLSX)

S1 Fig (A) Sampling design to evaluate the environment, demographic and landscape features at sites used to evaluate the removal of *Acca sellowiana* fruit by vertebrate fauna (mammals and birds) in the subtropical Atlantic Forest highlands, Brazil; and (B) Evidences of *Acca sellowiana* domestication via management within sites: Where: (B1) Tree with signals of pruning near to old rural propriety (S1); and (B2) Trees without signals of domestication (native) within the site S4.
(PDF)

S2 Fig PCA analysis to environmental, landscape, anthropization and *Acca sellowiana* density in four sites in the subtropical Atlantic Forest highlands, Brazil. Where: A: altitude (m.a.s.l.); AD: distance from nearest adult Feijoa (m); AC: focal-individual canopy area (m^2^); WA: distance from water bodies (m); OP: distance from open area (m); FD: distance from nearest fragment (m); FS: fragment/lowland size (ha); DO: canopy coverage (%); DT: diameter of nearest tree (m); HT: height of nearest tree (m); AT: distance from nearest tree (m); DS: diameter of nearest shrub (m); HS: height of nearest shrub (m); AS: distance of nearest shrub; LI: litterfall coverage (%); HE: herbaceous coverage (%); IN: inclination (°); IH: human presence intensity; IG: cattle intensity; Dom.: proportion of Feijoa trees with evidence of domestication within sites.
(PDF)

S3 Fig *Acca sellowiana* productivity (N and biomass) into four sampling sites and two temporal replicas in the subtropical Atlantic Forest highlands, Brazil. (PDF)

S4 Fig Rarefaction curve (with a confidence interval of 95% in gray) for all independent records of mammals and birds species (except exotic) during evaluation of fruit removal from *Acca sellowiana* in three sites in subtropical Atlantic Forest highlands, Brazil. Independent records were adjusted by sampling effort (camera-trap/day/replica). S1 and S2: São Joaquim National Park; and S4: RPPN Leão da Montanha.
(PDF)

S5 Fig (Left) Interaction between Cricetidae, *Eira barbara*, *Cerdocyon thous*, *Nasua nasua* and *Didelphis aurita* with *Acca sellowiana* fruits. (Right) Interaction between *Aramides saracura*, *Penelope obscura*, *Pyrrhura frontalis*, *Turdus rufiventris* and *Cyanocorax caeruleus* with Feijoa fruits in the subtropical Atlantic Forest highlands, Brazil.
(JPG)

S6 Fig Topology of the total network by the site for years 2015 (above) and 2016 (below) to evaluate the removal of *Acca sellowiana* fruit by vertebrate fauna (mammals and birds) in the subtropical Atlantic Forest highlands, Brazil. A: bipartite network considering 2015; B: modular network considering 2015; C: bipartite network considering 2016; D: modular network considering 2016. S1 and S2: São Joaquim National Park; S3: surrounding of RPPN Grande Floresta das Araucárias; and S4: RPPN Leão da Montanha.
(PDF)

S7 Fig (A) Network by groups, behavior, stratum, time and circadian period in TR1 (2015); and (B) Network by groups, behavior, stratum, time and circadian period in TR3 (2016) obtained in four sampling sites used to evaluate the removal of *Acca sellowiana* fruit by vertebrates in the subtropical Atlantic Forest highlands, Brazil. In this order: mammals, birds, endozoochory, sinzoochory, upper stratum, bottom stratum, early fruiting (< 30 days), end of fruiting (> 30 days), day and night.
(PDF)

S1 Movie Compiled video of frugivory in *Acca sellowiana* in the subtropical Atlantic Forest highlands, Brazil. (MP4)
