# Supplementary material for: The ecological footprint of Acca sellowiana domestication maintains the residual vertebrate diversity in threatened highlands of Atlantic Forest
Source: PLoS One. 2018 Apr 4;13(4):e0195199. doi: 10.1371/journal.pone.0195199 (PMC5884537; doi:10.1371/journal.pone.0195199)
Supplement: S1 Fig — (A) Sampling design to evaluate the environment, demographic and landscape features at sites used to evaluate the removal of Acca sellowiana fruit by vertebrate fauna (mammals and birds) in the subtropical Atlantic Forest highlands, Brazil; and (B) Evidences of Acca sellowiana domestication via management within sites: Where: (B1) Tree with signals of pruning near to old rural propriety (S1); and (B2) Trees without signals of domestication (native) within the site S4. (PDF) [file pone.0195199.s003.pdf]

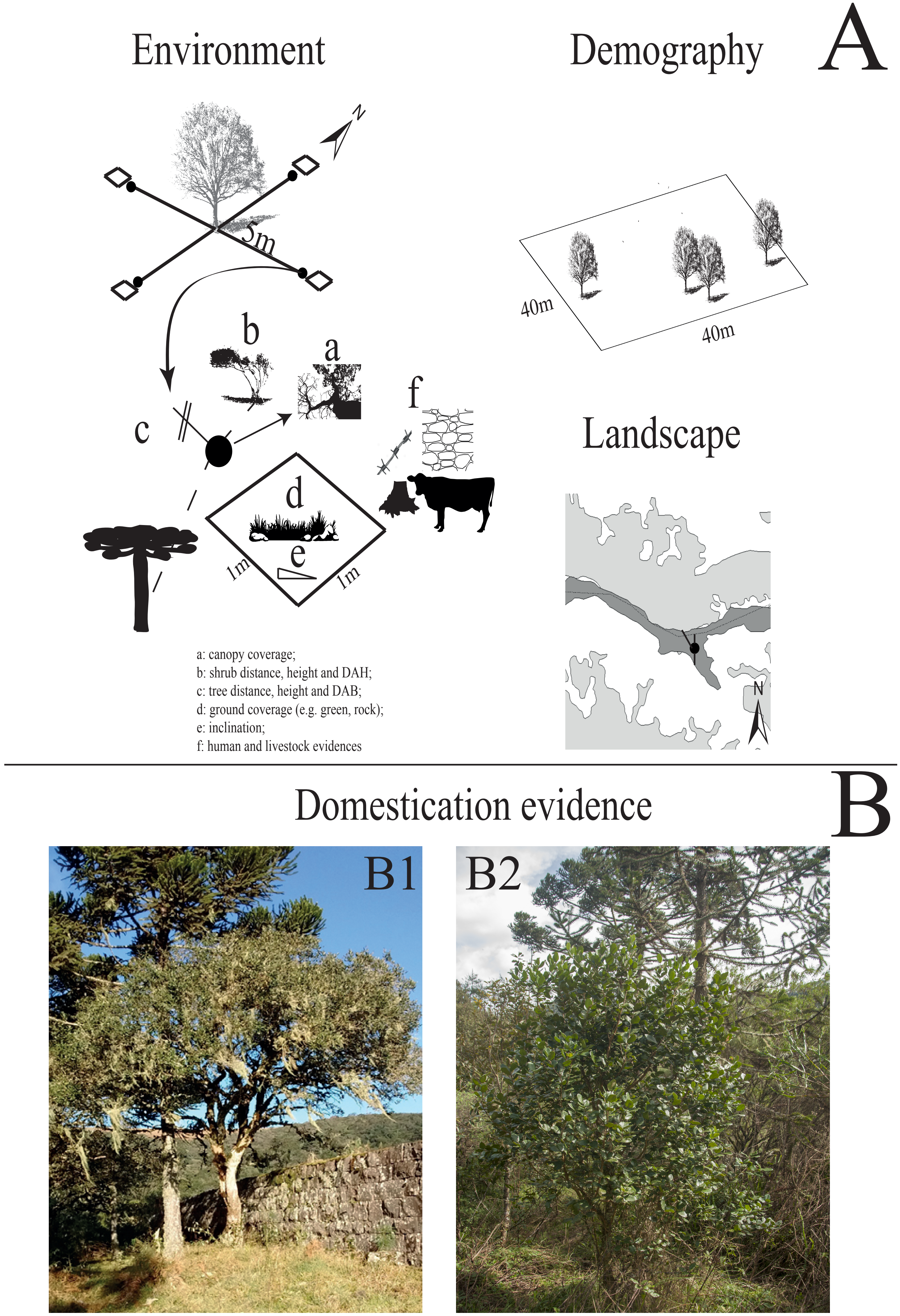

Environment
Demography
A
N
5m
40m
40m
b
a
f
c
Landscape
d
e
1m
1m
N
a: canopy coverage;
b: shrub distance, height and DAH;
c: tree distance, height and DAB;
d: ground coverage (e.g. green, rock);
e: inclination;
f: human and livestock evidences
Domestication evidence
B
B1
B2
