# Supplementary figures and images for: The ecological footprint of Acca sellowiana domestication maintains the residual vertebrate diversity in threatened highlands of Atlantic Forest
Source: PLoS One. 2018 Apr 4;13(4):e0195199. doi: 10.1371/journal.pone.0195199 (PMC5884537; doi:10.1371/journal.pone.0195199)

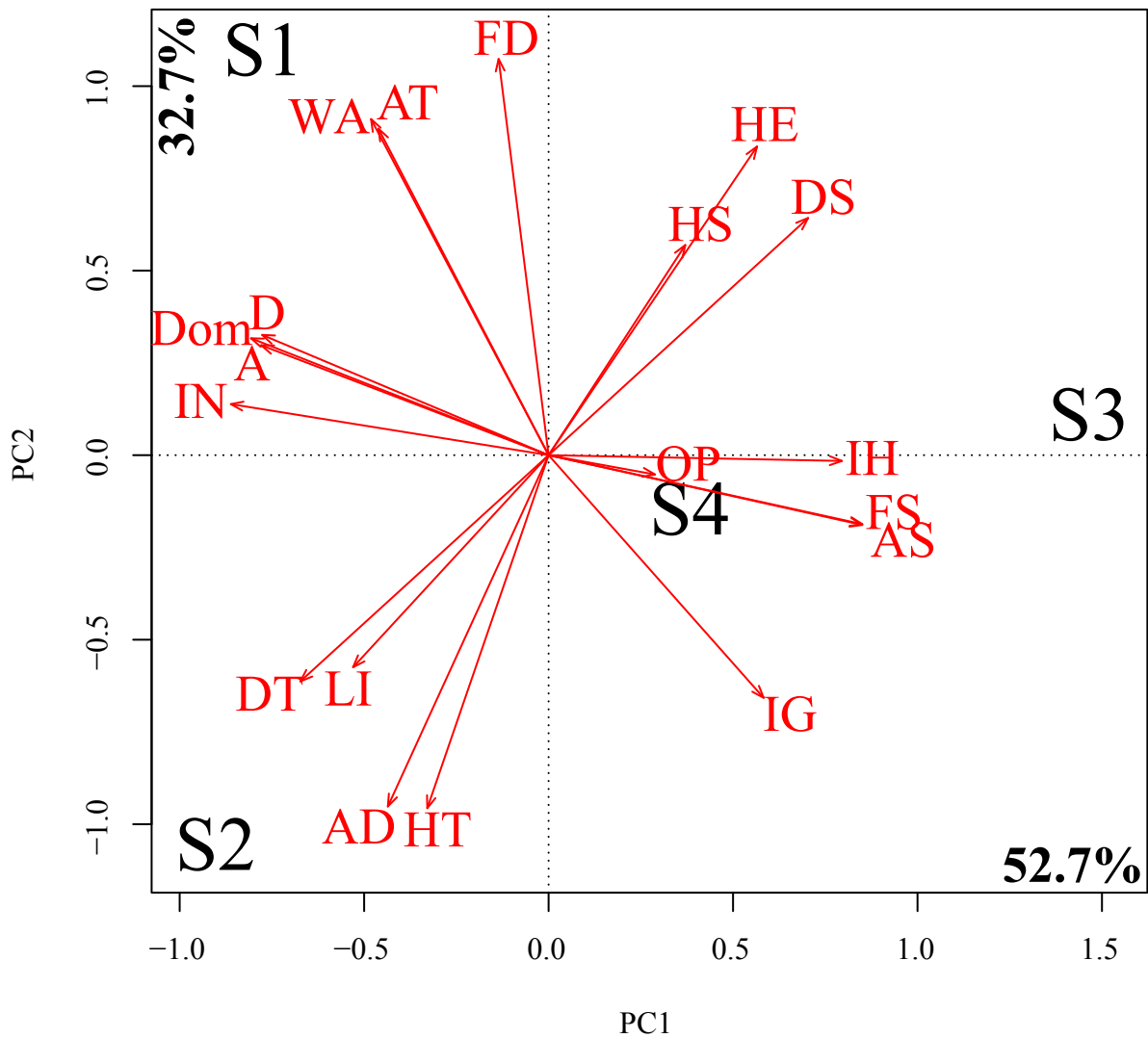

Supplement: S2 Fig — Where: A: altitude (m.a.s.l.); AD: distance from nearest adult Feijoa (m); AC: focal-individual canopy area (m2); WA: distance from water bodies (m); OP: distance from open area (m); FD: distance from nearest fragment (m); FS: fragment/lowland size (ha); DO: canopy coverage (%); DT: diameter of nearest tree (m); HT: height of nearest tree (m); AT: distance from nearest tree (m); DS: diameter of nearest shrub (m); HS: height of nearest shrub (m); AS: distance of nearest shrub; LI: litterfall coverage (%); HE: herbaceous coverage (%); IN: inclination (°); IH: human presence intensity; IG: cattle intensity; Dom.: proportion of Feijoa trees with evidence of domestication within sites. (PDF) [file pone.0195199.s004.pdf]

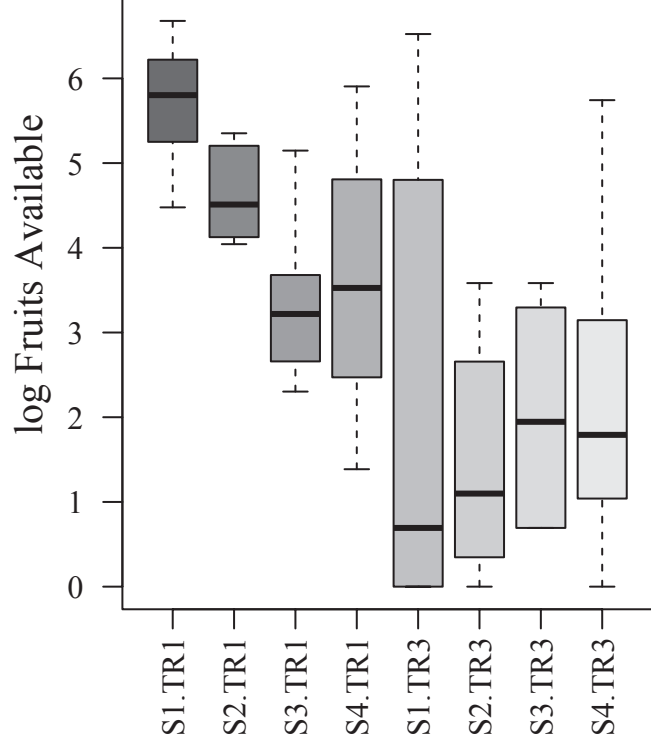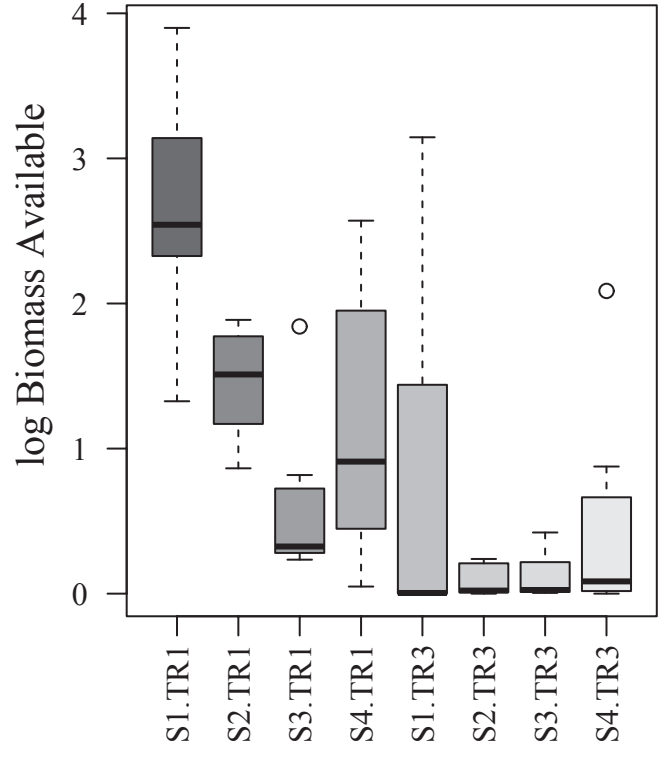

Supplement: S3 Fig — (PDF) [file pone.0195199.s005.pdf]

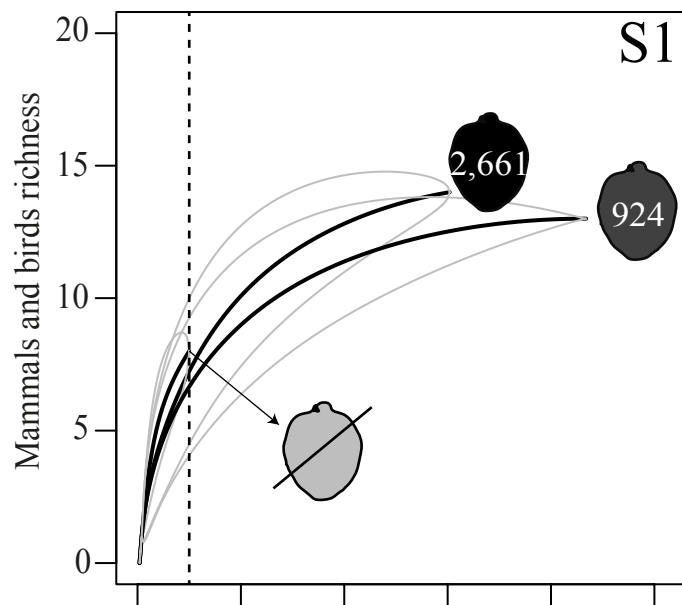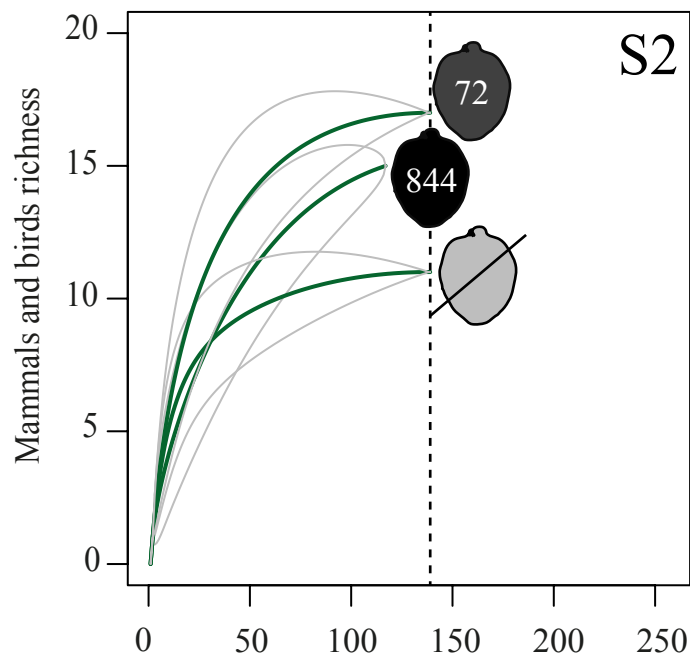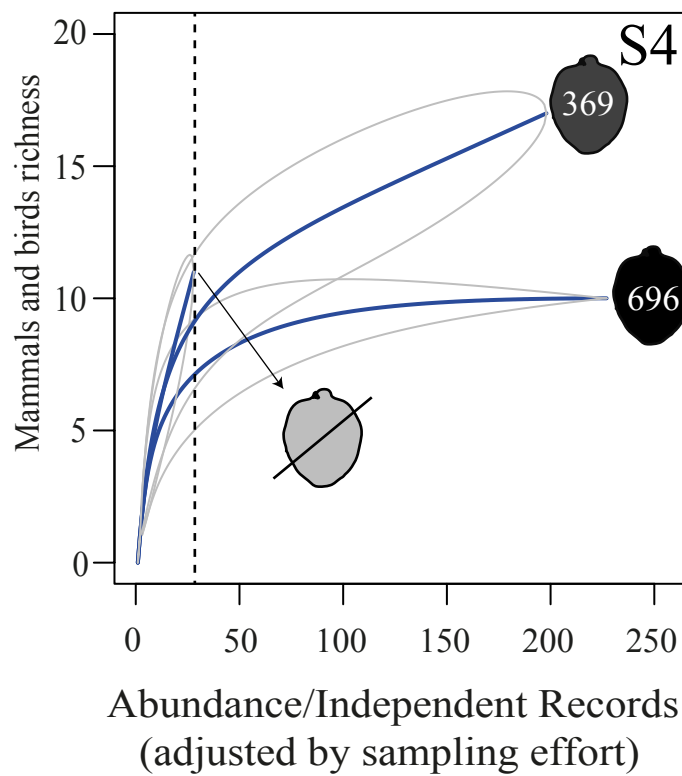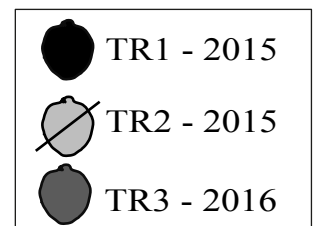

Supplement: S4 Fig — Independent records were adjusted by sampling effort (camera-trap/day/replica). S1 and S2: São Joaquim National Park; and S4: RPPN Leão da Montanha. (PDF) [file pone.0195199.s006.pdf]

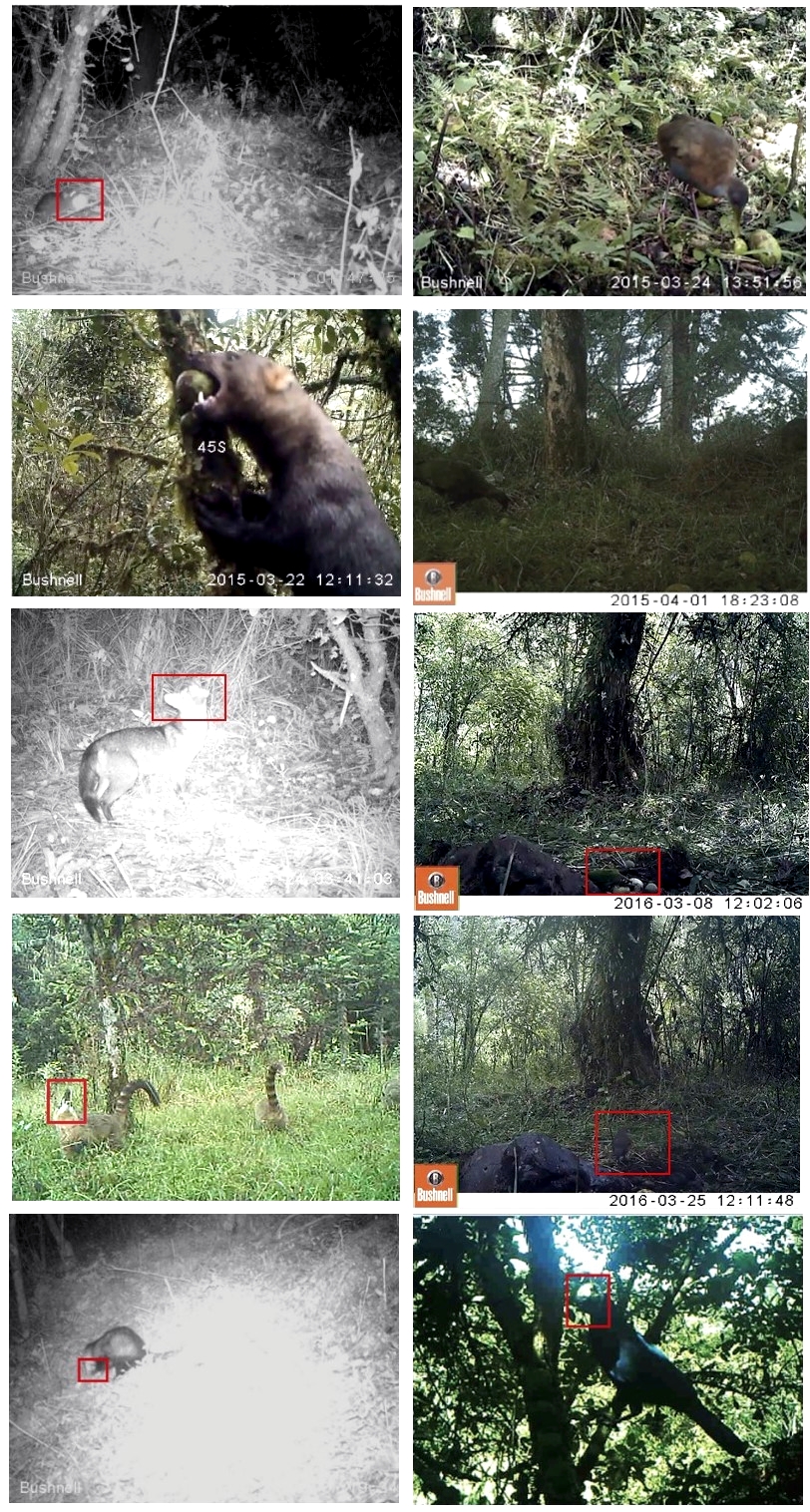

Supplement: S5 Fig — (Left) Interaction between Cricetidae, Eira barbara, Cerdocyon thous, Nasua nasua and Didelphis aurita with Acca sellowiana fruits. (Right) Interaction between Aramides saracura, Penelope obscura, Pyrrhura frontalis, Turdus rufiventris and Cyanocorax caeruleus with Feijoa fruits in the subtropical Atlantic Forest highlands, Brazil. (JPG) [file pone.0195199.s007.jpg]

TR1: 2015

A

B

4,514

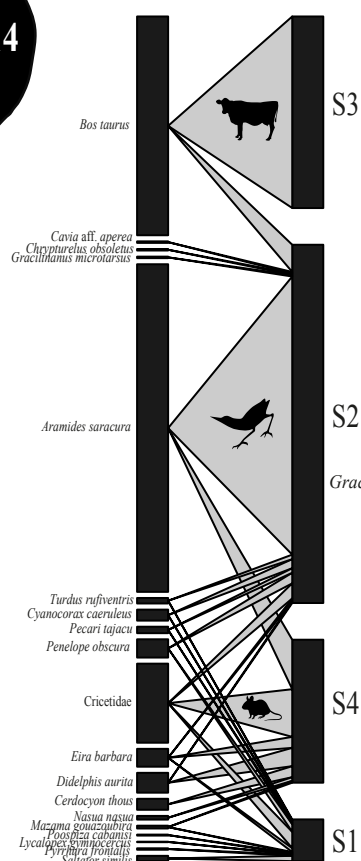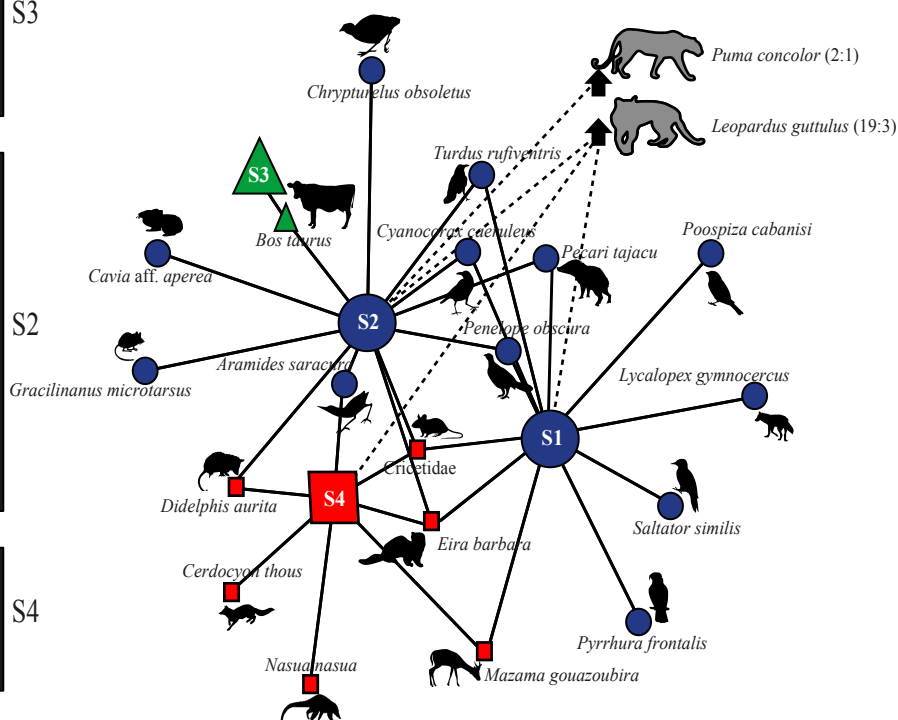

TR3: 2016

C

D

1,461

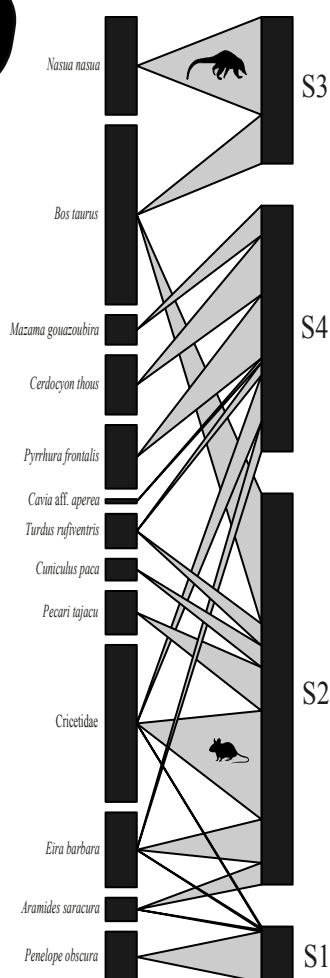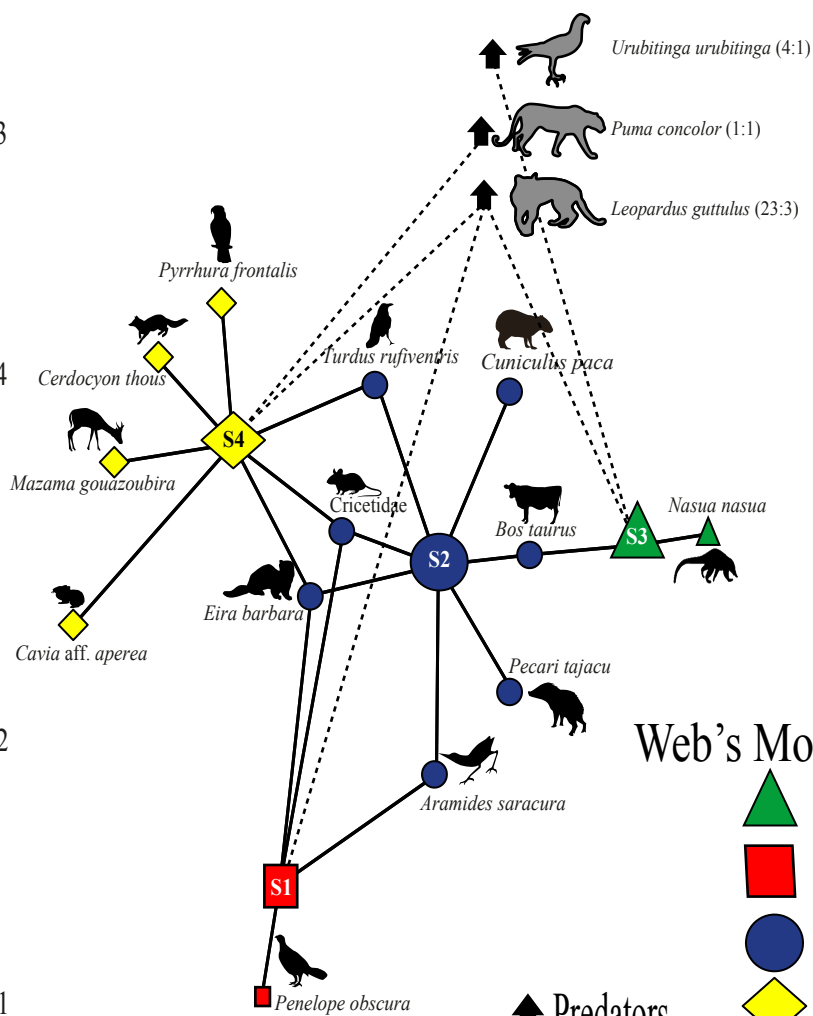

Web's Modules

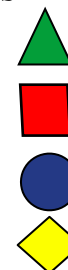

↑ Predators

Supplement: S6 Fig — A: bipartite network considering 2015; B: modular network considering 2015; C: bipartite network considering 2016; D: modular network considering 2016. S1 and S2: São Joaquim National Park; S3: surrounding of RPPN Grande Floresta das Araucárias; and S4: RPPN Leão da Montanha. (PDF) [file pone.0195199.s008.pdf]
