# Supplementary material for: The ecological footprint of Acca sellowiana domestication maintains the residual vertebrate diversity in threatened highlands of Atlantic Forest
Source: PLoS One. 2018 Apr 4;13(4):e0195199. doi: 10.1371/journal.pone.0195199 (PMC5884537; doi:10.1371/journal.pone.0195199)
Supplement: S7 Fig — (A) Network by groups, behavior, stratum, time and circadian period in TR1 (2015); and (B) Network by groups, behavior, stratum, time and circadian period in TR3 (2016) obtained in four sampling sites used to evaluate the removal of Acca sellowiana fruit by vertebrates in the subtropical Atlantic Forest highlands, Brazil. In this order: mammals, birds, endozoochory, sinzoochory, upper stratum, bottom stratum, early fruiting (< 30 days), end of fruiting (> 30 days), day and night. (PDF) [file pone.0195199.s009.pdf]

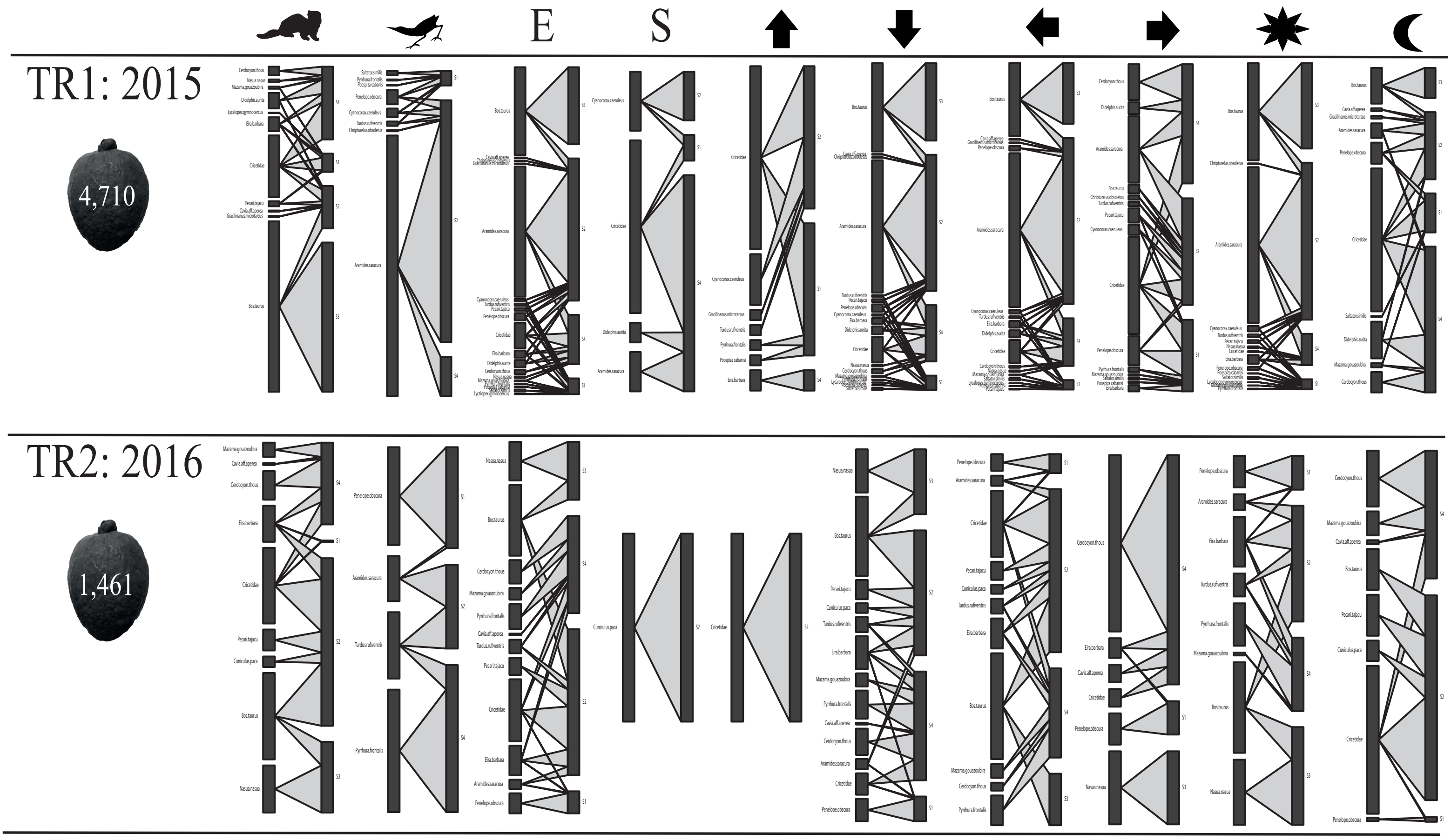
E
S
TR1: 2015
4,710
TR2: 2016
1,461
